# Dynamic Anchoring of Peptides to the Extracellular Matrix Enabled by Boronic Acid

**DOI:** 10.1002/cbic.202500569

**Published:** 2025-11-24

**Authors:** Martin Mergenthaler, Eduardo Merino Asumendi, Andreas Inho Höpfel, Marcus Gutmann, Lorenz Meinel, Tessa Lühmann

**Affiliations:** ^1^ Institut for Pharmacy and Food Chemistry Universität Würzburg Am Hubland 97074 Würzburg Germany; ^2^ Helmholtz Center for Infection Research (HZI) Helmholtz Institute for RNA‐Based Infection Research (HIRI) 97080 Wuürzburg Germany

**Keywords:** boronic acid, diol‐rich glycans, extracellular matrix, myostatin inhibitor, reversible binding

## Abstract

Given the extracellular matrix (ECM) essential role in tissue development, maintenance, and repair, exploiting its glycan structures offers new opportunities for dynamic drug storage and targeted delivery of peptides via injectable solutions. This study investigates the reversible, pH–driven interaction between boronic acid‐functionalized compounds and diol‐rich glycans within cell‐derived decellularized extracellular matrices (CDMs). An N‐terminally functionalized model peptide was produced using carboxy‐phenyl‐boronic acid (CPBA), and its pH‐dependent binding to the diol‐rich structures of CDMs was demonstrated. A CPBA‐conjugated myostatin inhibitor maintained its full potency as a prerequisite for future therapeutic application. The findings suggest N‐terminally boronic acid‐modified peptides as drug candidates for targeted delivery and storage to diol‐rich glycans of the natural ECM.

## Graphical abstract

Peptides are developed for reversible binding to sugar‐rich structures (glycans) in cell‐derived extracellular matrix models using boronic acid chemistry. By attaching a carboxy‐phenyl‐boronic acid group to the N‐terminus of peptides, a modified myostatin inhibitor is synthesized that retaines biological activity comparable to standard inhibitors. This modification may improve how these peptides interact with tissues, offering potential benefits for drug delivery in future pharmacological studies.

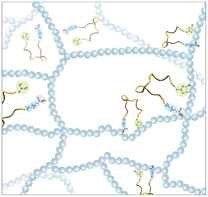

## 1 Introduction

**Scheme 1:**
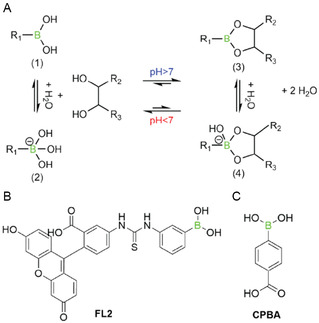
A) Equilibrium formation of a boronic ester (3, 4) from boronic acid (1, 2) and a 1,2‐diol, above and below pH 7 in water. B) Fluorescein boronic acid (FL2). C) 4‐Carboxy‐phenyl‐boronic acid (CPBA).

Boronic acids represent the most extensively studied class of organoboronic compounds, widely utilized in hydrogel design for drug delivery,^[^ ^1^ ^]^ the development of sensors,^[^ ^2^ ^]^ and nucleotide transport^[^ ^3^ ^]^ owing to their distinctive ability to form reversible covalent bonds with 1,2‐diols and other nucleophilic species (**Scheme** **1**A).

Due to their chemical properties, boronic acids can form reversible esters with Lewis bases. Natural binding partners include hydroxyl groups present in carbohydrates, nucleic acids, and amino acids.^[^ ^4^ ^,^ ^5^ ^]^ In particular, the interaction between boronic acids and 1,2‐diols is considered one of the strongest single‐pair reversible group interactions in an aqueous environment.^[^ ^6^ ^]^ Accordingly, boronic acid integration into recombinant proteins has been performed by various methods, including site‐specific click chemistry,^[^ ^7^ ^]^ genetic engineering by amber codon expansion,^[^ ^8^ ^]^ and by providing dehydroalanines (DHA) during recombinant protein synthesis to site‐specifically form boronalanines.^[^ ^9^ ^]^ Moreover, a benzoxaborole containing an NHS‐sensitive linker was effective for traceless intracellular delivery of the green fluorescent protein.^[^ ^10^ ^]^ Synthetic routes of boronic acid integration in peptides are currently extensively explored and comprise late‐stage hydroboration of peptide alkynes on‐resin,^[^ ^11^ ^]^ N‐terminal modification with vinylboronic acids,^[^ ^12^ ^]^ and thiol‐ene click chemistry for site‐specific cysteine modification.^[^ ^13^ ^]^

Particularly, the pH‐sensitivity of boronic acid‐decorated biomacromolecules has facilitated the controlled release from hydrogels and into endosomal compartments.^[^ ^1^ ^]^ Interestingly, the delivery and storage of boronic acid‐conjugated drugs to glycoproteins of the extracellular matrix (ECM) have, to date, been underexplored, although they provide suitable diol‐rich glycan structures for interaction. The cell surrounding ECM is essential for tissue development, maintenance, and repair by regulating cell behavior, migration, differentiation, proliferation, and survival. ECM components and cell‐derived decellularized extracellular matrices (CDMs) serve as bioengineered 3D microenvironments, offering mechanical support, promoting cell adhesion and migration, modulating cell activity, and storing growth factors. As a result, ECM‐based scaffolds are already being evaluated in clinical trials. A recent report detailed the potential of live‐imaging of ECM components in various tissues using a boronic acid‐modified small‐molecular fluorophore.^[^ ^14^ ^]^

Nature achieves protein and peptide depots by catalyzing covalent bonds with the ECM of tissues.^[^ ^15^ ^]^ We have previously demonstrated bioorthogonal modification of ECM components in CDMs by metabolic glycoengineering to introduce clickable glycan moieties^[^ ^16^ ^]^ and by enzymatic modification deploying transglutaminase (TGase) to form covalent *ε*‐(*γ*‐glutamyl)lysine isopeptide bonds.^[^ ^17^ ^]^

In this study, we aimed at expanding the dynamic interplay of boronic acids with diol‐rich glycans toward decoration of natural ECM components as a sustainable peptide drug depot from injectable solutions. First, we demonstrated reversible binding of a fluorescein boronic acid compound to diol‐rich glycans in cell‐derived ECM models, modulated by pH changes. Boronic acid conjugation was further employed for N‐terminal functionalization of a short model peptide and analyzed for binding to different monosaccharides using an Alizarin Red S (ARS) competition assay. Finally, we transferred boronic acid modification to a therapeutic anti‐myostatin (MI) peptide, enabling specific binding to 1,2‐diol moieties while preserving the peptide's bioactivity as a prerequisite for future biomedical applications.

## 2 Results and Discussion

### 2.1 Boronic Acid Interactions with ECM Components in Different pH Environments

We first asked if the commercially available dye fluorescein boronic acid (FL2; Scheme 1B) can reversibly interact with ECM‐derived components by toggling between physiological pH and acidic pH, such as present in lysosomes or inflamed tissue sites. We generated CDMs from murine NIH‐3T3 fibroblasts in the presence of 50 µg/mL ascorbic acid to stimulate collagen fibrillogenesis, following established protocols.^[^ ^16^ ^]^ We then chose to analyze the ECM glycoprotein fibronectin as a counterstain, as it possesses multiple diol‐rich glycosylation sites.^[^ ^16^ ^]^ After immunostaining for fibronectin, the CDMs were incubated with FL2 in PBS (7.4) for 1 h, and then washed with buffers of three different pH values (pH 4.1, pH 7.4, and pH 9.4).

**Figure 1:**
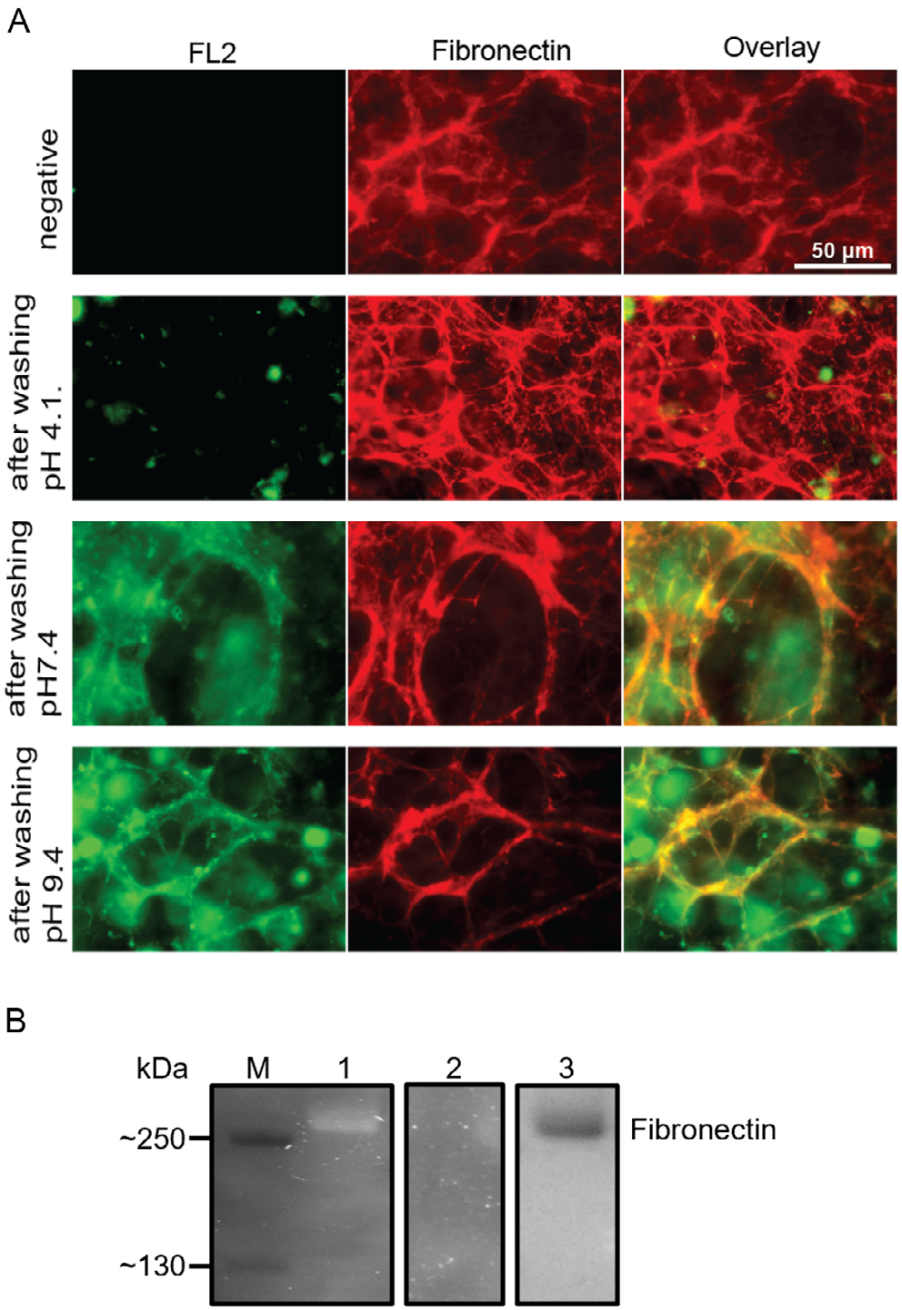
A) Fluorescent microscopy of CDM. Green channel: Staining with 10 μM FL2 is shown. Red channel: Staining with a primary antibody (rabbit; Anti‐fibronectin) and secondary antibody (Anti‐Rabbit IgG, CFTM633) is shown. Third channel: Both channels are merged. The negative control was only stained with the antibodies. Every CDM with FL2 was washed several times with either pH 4.1, pH 7.4, or pH 9.4. B) SDS‐PAGE of fibronectin incubated with FL2 under different pH conditions. (1) FL2 incubation at pH 7.4. (2) FL2 incubation at pH 4.1. (3) Coomassie stain for fibronectin. M = molecular standard.

FL2 was strongly present on defined fibrillar components when exposed to neutral or basic pH and colocalized with the fibronectin counterstain (**Figure** **1**A). At acid pH, fluorescence of FL2 was absent, indicating dissociation from the CDM scaffold.

The binding of ARS to fibronectin was confirmed at physiological pH after gel electrophoresis with a distinct fluorescence at 280 kDa (Figure 1B). For quantification, fibronectin was immobilized on Enzyme‐Linked Immunosorbent Assay (ELISA) plates before incubation with FL2. Surfaces were similarly treated with acidic, neutral, and basic conditions, and the remaining fluorescence of FL2 was quantified with each washing step (Figure S1, Supporting Information). The Fluorescence intensity was highest for neutral and basic buffer conditions and dropped significantly when acidic buffer conditions were applied (Figure S1D, Supporting Information).

Both results demonstrate the efficacy of the phenyl boronic acid moiety, derived from FL2, in binding to fibronectin at physiologically relevant conditions and in releasing from the glycoprotein in response to a more acidic environment.

### 2.2 Synthesis and Performances of Boronic Acid‐Modified Peptides

**Figure 2:**
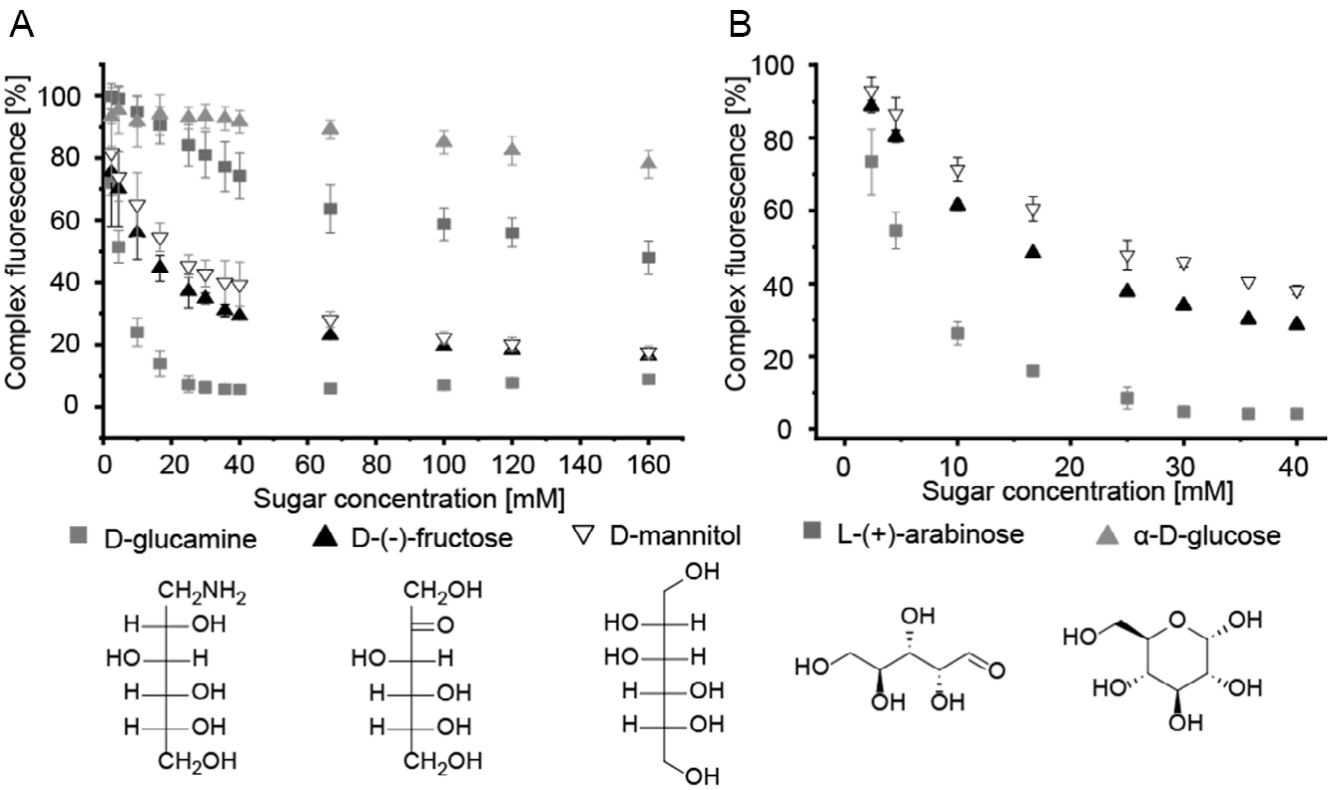
A) Fluorescence decay at 572 nm measured in ARS competition assay with CPBA and several monosaccharides. B) Fluorescence decay at 572 nm was measured in an ARS competition assay with CPBA‐labeled peptide and several monosaccharides. Data are shown as mean ± STDEV (*n* = 3).

As next, we aimed to exchange the fluorophore against a TGase recognition peptide derived from the *α*2‐antiplasmin inhibitor.^[^ ^18^ ^]^ This peptide sequence, NQEQVSPL, was N‐terminally modified on‐resin by coupling 4‐carboxy‐phenyl‐boronic acid (CPBA; Scheme 1C, Figure S2, Supporting Information). Following the coupling, a washing step, a deprotection‐cleavage reaction, and purification, the CPBA‐labeled NQEQVSPL peptide (CPBA‐peptide) was obtained in 95% purity (Figure S2, Supporting Information). Affinity toward different monosaccharides was tested quantitatively by an Alizarin Red S (ARS) competition assay. ARS spectroscopic properties change upon the formation of a boronic ester with a boronic acid, resulting in fluorescence.^[^ ^4^ ^,^ ^19^ ^]^ Additionally, the absorbance maximum of ARS shifts to shorter wavelengths upon ester formation, resulting in a color shift from red to orange, which facilitates the recognition of the formed bond (Figures S3 and S4, Supporting Information). The affinity of aryl boronic acid to 1,2‐diols is known to be dependent on modifications of the underlying benzene. Therefore, the CPBA 1,2‐diol interaction may be changed after N‐terminal peptide‐coupling.^[^ ^4^ ^]^ For this reason, the ARS competition assay was done for both molecules, CPBA as a reference and CPBA‐peptide to study binding to model saccharides and a polyalcohol as determined by the equilibrium constants *K*_eq_ toward ARS at physiological pH 7.4 (**Figure** **2**, **Table** **1** **,** Figures S5–S7, Supporting Information).

**Table 1:** Association constants *K*_eq_ at pH 7.4 in a 0.1 M phosphate buffer (pH 7.4). Values are the average of triplicate runs, rounded to two significant figures.

	CPBA [*K*_eq_] [mM^−1^]	CPBA‐peptide [*K*_eq_] [mM^−1^]
ARS	1.46 ± 0.3	2.35 ± 0.1
D‐glucamine	2.09 ± 0.6	3.87 ± 0.7
D‐(‐)‐fructose	0.11 ± 0.0	0.29 ± 0.0
D‐mannitol	0.08 ± 0.0	0.22 ± 0.0
L‐(+) arabinose	–	–
*α*‐D‐glucose	–	–

Binding constants of boronic acids and diols have been previously linked to their pK_A_ and pH environment, with phenylboronic acid (pK_A_ = 8.8) showing a binding optimum with ARS at a pH of 7 (1.1 mM^−1^).^[^ ^20^ ^]^

In our hands, the interaction of CPBA with D‐glucamine was found to be strongest (*K*_eq_ = 2.09 mM^−1^) and very weak for L‐(+)‐arabinose and *α*‐D‐glucose (*K*_eq_ = not determinable). The association constant with ARS and the CPBA‐peptide was 1.6‐fold higher than that toward unconjugated CPBA, demonstrating some influence of the N‐terminal peptide attachment on the phenyl‐boronic acid moiety for the herein studied 1,2‐diol affinity with monosaccharides.

We next examined whether the N‐terminally modified boronic acid–modified NQEQVSPL‐peptide shows pH‐dependent binding properties to CDMs. To enable fluorescence detection, a CPBA–NQEQVSPL–PEG_6_–azidohomoalanine variant was synthesized and coupled via strain‐promoted alkyne‐azide cycloaddition (SPAAC) to DBCO–PEG_4_–5/6‐carboxyrhodamine 110, resulting in >95% purity (Figure S8, Supporting Information). The hydrophilic PEG_6_ linker within the peptide structure was crucial to ensure sufficient water solubility. CDMs were generated and counterstained as described above and incubated with CPBA–NQEQVSPL–5/6‐carboxyrhodamine 110 as outlined above for FL2 (Figure S9, Supporting Information). The boronic‐acid functionalized peptide interacted with glycan structures of the ECM similarly to the previously tested FL2 and exhibited comparable pH‐dependent binding behavior. However, the binding at pH 7.4 was quantitatively weaker than that observed for FL2, likely due to partial steric shielding of the boronic acid within the peptide scaffold and the different pKa of the arylboronic acid moiety, which likely influenced the fraction of the reactive boronate species at physiological pH.

### 2.3 Design and Synthesis of Boronic Acid‐Modified Myostatin Inhibitor (MI) Peptides

**Figure 3:**
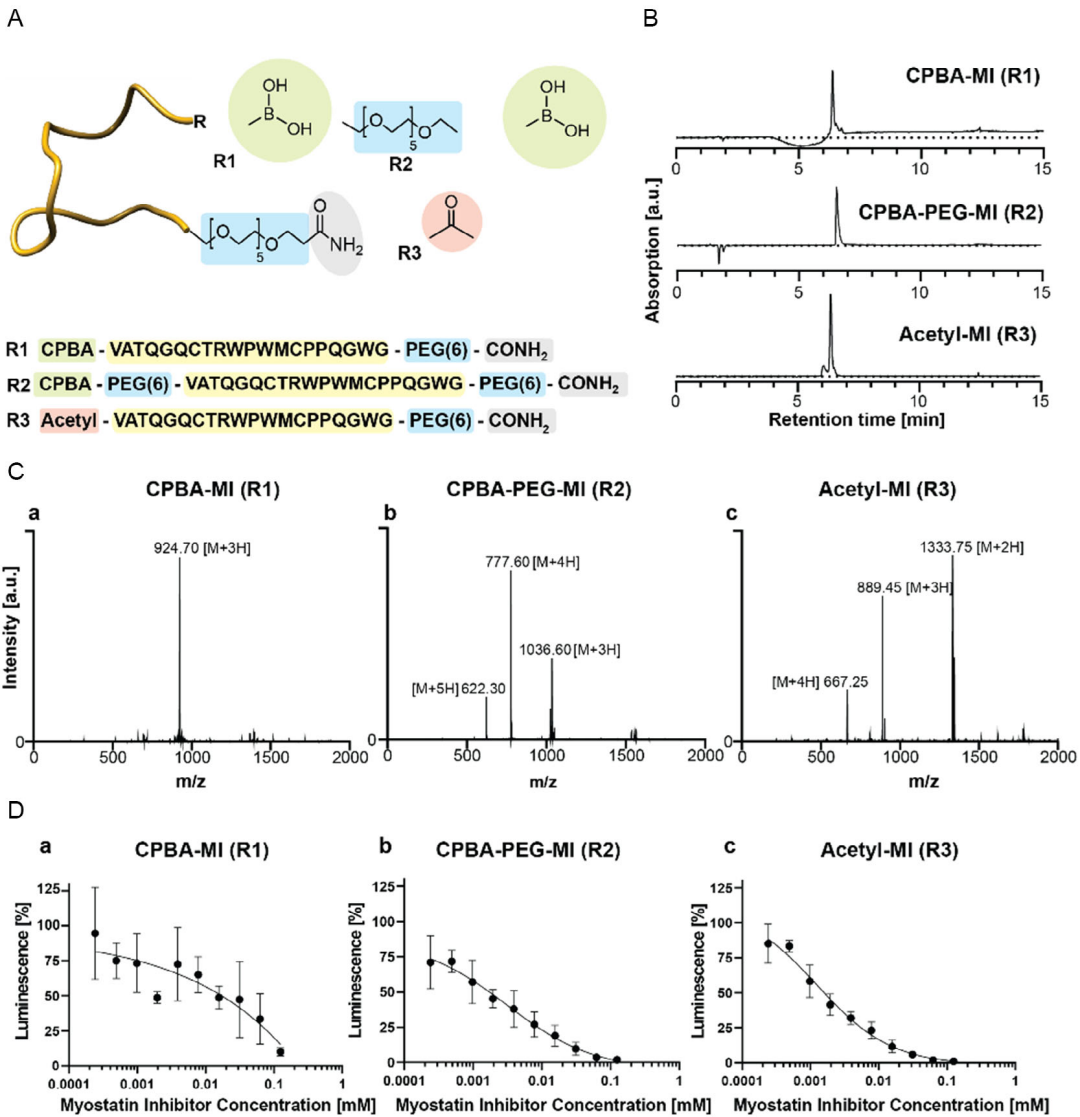
A) MI design and corresponding peptide sequence. B) RP‐HPLC analytics and LC‐MS ‐spectra C) of synthesized MIs for identification and purity. (a) observed average mass: 924.70 Da [M + 3H]^+3^; expected average mass 923.99 Da [M + 3H]^3+^. (b) Observed average mass: 1036.60 Da [M + 3H]^+3^, expected average mass 1035.79 Da [M + 3H]^+3^. (c) Observed average mass: 1333.75 Da [M + 2H]^2+^; expected average mass 1333.05 Da [M + 2H]^2+^. D) Myostatin inhibition by different MI peptides as determined via SMAD luciferase reporter with HEK SBE cells. 100 % activity corresponds to 4 nM myostatin. Data are shown as mean ± STDEV (*n* = 3).

To demonstrate N‐terminal modification and function of boronic acid within a therapeutic peptide, we selected the 3 kDa myostatin inhibitor (MI). Myostatin, a key regulator of muscle atrophy and tissue repair, signals through heterodimeric activin receptors (ActRIIB and ALK4/5) to inhibit myogenesis.^[^ ^21^ ^]^ While it has been extensively studied in the context of muscle wasting, emerging evidence indicates a role in modulating wound healing by attenuating fibrosis and inflammatory responses.^[^ ^22^ ^]^ Current therapeutic strategies primarily target ActRIIB via decoy receptors or neutralizing antibodies to prevent receptor activation.^[^ ^23^ ^]^ The MI peptide with the sequence VATQGQCTRWPWMCPPQGWG and its modifications thereof were synthesized via automated SPPS as previously detailed.^[^ ^17^ ^,^ ^24^ ^]^ The N‐terminus was modified with CPBA (vide supra) and acetylated for a control (nonglycan binding) MI peptide (**Figure** **3**A).

**Figure 4:**
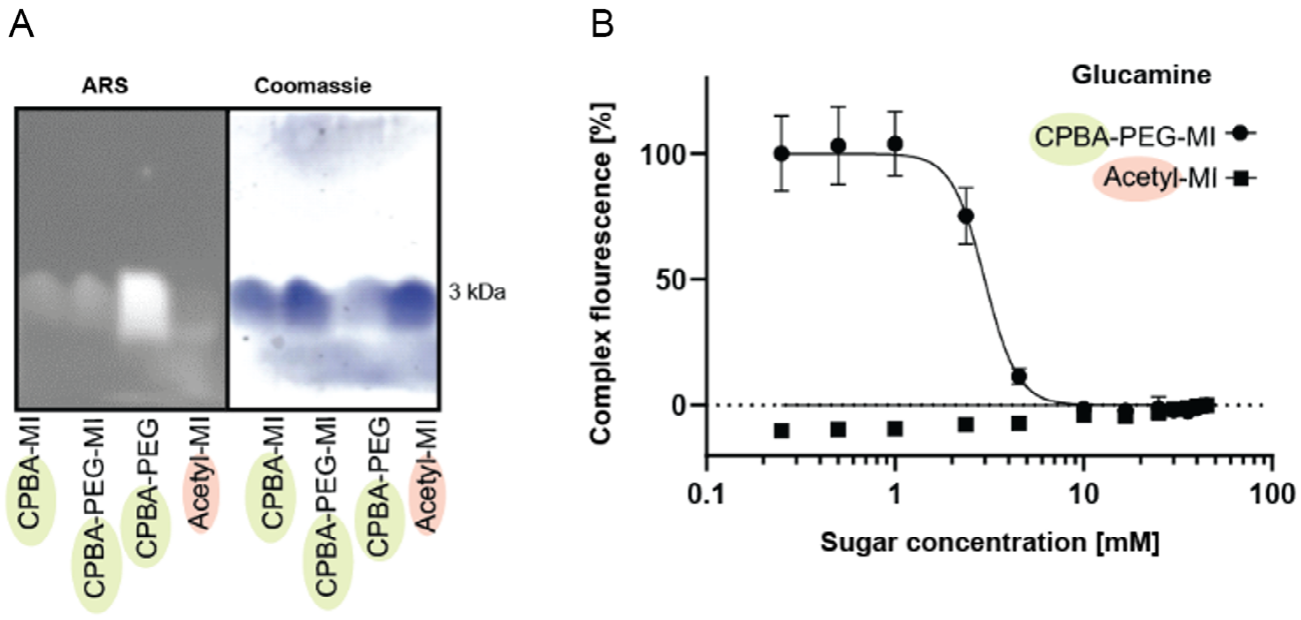
A) Tricine gel electrophoresis with ARS and subsequent Coomassie staining. B) Fluorescence decay at 572 nm measured in an ARS competition assay with CPBA‐PEG‐MI and the negative control Acetyl‐MI in the presence of rising concentrations of D‐glucamine as a model saccharide at pH 7.4. Data are shown as mean ± STDEV (*n* = 3).

To achieve better solubility and accessibility of the boronic acid moiety, a small PEG linker was interpositioned between the peptide and the CPBA structure (CPBA‐PEG‐MI). Purity and identity of all MI‐peptides were assessed by RP‐HPLC and LC‐MS/MS analysis (Figure 3B,C). The inhibition of all three MI peptides by myostatin was tested using a reporter gene luciferase assay in HEK293 cells, which monitored SMAD phosphorylation in response to varying myostatin concentrations, as described.^[^ ^24^ ^]^ For the CPBA‐MI‐peptide, which was directly coupled to CPBA at the N‐terminus, a high variation in luminescence was recognized (Figure 3D), potentially due to the poor water‐solubility and formation of CPBA‐MI‐peptide‐aggregates (data not shown). The other two MI peptides displayed both a dose‐dependent inhibitory effect on myostatin‐induced SMAD phosphorylation, with IC_50_ values in the μ‐molar range (IC_50_ CPBA‐PEG‐MI = 0.0015 mM; IC_50_ Acetyl‐MI = 0.0017 mM) in accordance to previously reported performances of MI inhibitors.^[^ ^24^ ^]^ As a next step, we analyzed the ability of CPBA‐modified MI‐peptides to interact with ARS and evolve fluorescence after tricine gel electrophoresis (**Figure** **4**A).

As a positive control, we used 4‐(dihydroxyboryl)benzoic acid coupled to the hydrophilic polymer PEG of 2000 Da, with a similar molecular weight. All boronic acid conjugated MI‐peptides and the boronic acid functionalized PEG displayed distinct fluorescence after ARS staining in contrast to the control peptide Acetyl‐MI. These results were confirmed by the shift of the absorbance maximum of ARS to shorter wavelengths upon ester formation of CBPA‐PEG‐MI and CBPA‐MI but not for Acetyl‐MI (Figure S10, Supporting Information). We continued to examine the binding of CBPA‐PEG‐MI to ARS in the presence of D‐glucamine, D‐(‐)‐fructose and D‐mannitol as a competition assay as outlined above (Figure 4B, Figure S11, Supporting Information). D‐glucamine showed the strongest interaction with CBPA‐PEG‐MI, with increasing monosaccharide concentrations in contrast to the nonbinding control Acetyl‐MI. D‐(‐)‐fructose, and D‐mannitol were both not able to displace ARS from the ARS–CPBA‐PEG–MI complex at the given sugar concentration (Figure S11, Supporting Information).

Upcoming studies will focus on evaluating the performance of the developed CBPA‐PEG‐MI peptide anchored to diol‐rich ECM components within arthritic joints. Ongoing in vitro investigations are assessing how CBPA‐PEG‐MI functionalized ECM influences signaling pathway activation and inhibits the differentiation of macrophages into osteoclasts. As a high‐affinity (micromolar) myostatin trap, the CBPA‐PEG‐MI peptide holds promise for addressing current pharmacokinetic limitations, particularly the short half‐life associated with small peptide inhibitors.^[^ ^25^ ^]^

## 3 Conclusion

This study demonstrated the reversible binding of a fluorescein boronic acid compound to diol‐rich glycans in cell‐derived ECM models, modulated by pH changes. To leverage this reversible interaction for therapeutic purposes, we designed N‐terminally functionalized peptides using carboxy‐phenyl‐boronic acid (CPBA). A CPBA‐conjugated myostatin inhibitor showed comparable potency to standard MI peptides and holds potential for future pharmacokinetic and pharmacodynamic investigations due to altered tissue binding properties. The synthetic approach outlined here provides a versatile platform for developing boronic acid‐modified peptides at the N‐terminus, with broader biomedical applications.

## 4 Experimental Section

4.1

4.1.1

##### Materials

The following chemicals were purchased from Sigma–Aldrich (Taufkirchen, Germany): N,N‐diisopropylcarbodiimide (DIC), ethyl cyanohydroxyiminoacetate (Oxyma), hydroxybenzotriazole (HOBt), trifluoroacetic acid (TFA), triisopropylsilane (TIS), N‐(*tert*‐butoxycarbonyl)‐L‐cysteine methyl ester 2,2′‐(ethylenedioxy)diethanethiol (DODT), acetic anhydride, Fmoc protected (L)‐amino acids, RPIM 1640 medium—high glucose, fetal calf serum (FCS), penicillin−streptomycin, 2,3,4,5,6–, dithiothreitol (DTT), trifluoroethanol (TFE), HPLC‐grade acetonitrile (ACN), Luciferase Assay Reagent, Goat Anti‐Rabbit IgG, CFTM633 Antibody (SAB4600141), Rabbit Anti‐Fibronectin Antibody (F3648) and carboxyphenyl‐boronic acid (CPBA). N,N‐Diisopropylethylamine (DIPEA) was purchased from Carl Roth GmbH + Co. KG (Karlsruhe, Germany). Fluorescein boronic acid (FL2) was from abcr GmBH (Karlsruhe, Germany), mPEG Boronic acid was from Creative PEGWorks (Durham, North Carolina), myostatin was from Bio‐techne (Minneapolis, Minesota). DBCO‐PEG4‐5/6‐Carboxyrhodamine 110 was from Jena Bioscience (Jena, Germany). Anti‐rabbit IgG (H + L), F(ab’)2 Fragment (Alexa Fluor 647 Conjugate) #4414 was from Cell Signaling (Leiden, Netherlands). Deionized water was obtained from our in‐house Merck Millipore water purification system (Darmstadt, Germany). All other chemicals used were of at least pharmaceutical grade.

##### Peptide Synthesis

NQEQVSPL peptides were synthesized following an automated solid‐phase peptide synthesis protocol with Rink Amide MBHA resins as reported.^[^ ^26^ ^]^ Briefly, every amino acid was coupled using DIC/Oxyma in DMF at 90 °C, while Fmoc‐deprotection was performed with 20% piperidine in DMF. N‐acetylation was performed manually with 75 µL of acetic anhydride and 135 µL DIPEA in 2 mL of DMF for 30 min at room temperature (RT). After sequential washing with DMF, DCM, and diethyl ether, the peptide was fully deprotected and cleaved from the resin by resuspending in 8 mL of TFA:DODT:TIS:H_2_O (92.5:2.5:2.5:2.5) for 3 h. For MI peptides, automated solid‐phase peptide synthesis was performed for all natural amino acids with modifications for histidine (50 °C; 10 min) and proline (double coupling at 50 °C for 10 min each). Coupling of Fmoc‐NH‐PEG(6)‐COOH was performed manually using a fivefold molar excess in DMF overnight.

The peptides were precipitated from the cleavage solution with diethyl ether and centrifuged three times, in which the supernatant was discarded and replaced with cold diethyl ether. The pellet was dried under an air flow.

##### Peptide Modification with CPBA

CPBA was coupled manually to the deprotected N‐terminal sites of the peptides. CPBA was dissolved in 3 ml DMF with DIC, DIPEA, and HOBt in a fivefold molar excess. The coupling was manually done twice for NQEQVSPL and three times for MI peptides at RT on a shaker for 2 h before washing and final deprotection.

##### Purification of Peptides

Peptides were purified on an Äkta pure FPLC system (Marlborough, MA, USA) with a Luna 10u C18 100A (250 × 21.2 mm) reversed‐phase column (Phenomenex Inc., Torrance, CA, USA) using a linear gradient of ACN and water with 0.1% TFA (v/v). UV‐absorbance was monitored at *λ* = 214 nm. The collected fractions were analyzed by LC−MS and HPLC. ACN and TFA were evaporated under nitrogen flow, and samples were freeze‐dried in a freeze dryer Alpha 1−4 (Martin Christ Gefriertrocknungsanlagen GmbH, Osterode, Germany). The lyophilized peptides were stored at −80 °C until use.

##### Peptide Modification with 5/6‐Carboxyrhodamine 110

Purified and lyophilized CPBA‐NQEQVSPL‐PEG(6)‐azidohomoalanine was dissolved in 30% (v/v) DMF in PBS and reacted with DBCO‐PEG4‐5/6‐Carboxyrhodamine 110, which was likewise prepared in 30% (v/v) DMF in PBS. The strain‐promoted azide–alkyne cycloaddition (SPAAC) was carried out for 2 h at RT, followed by incubation for 24 h at 4 °C, using a 1.2‐fold molar excess of DBCO‐PEG4‐5/6‐Carboxyrhodamine 110. The resulting CPBA‐NQEAVSPL‐5/6‐Carboxyrhodamine 110 peptide was subsequently purified and freeze‐dried as described above (vide supra).

##### Liquid Chromatography Mass Spectrometry (LC‐MS/MS)

Analysis of peptides was performed using a Single Quadrupole system, consisting of an LC20AB liquid chromatograph, an SPD‐20A UV/VIS detector, and an LC‐MS 2020 (Shimadzu Scientific Instruments, Columbia, MD, USA). To separate samples by LC, a Synergi 4 μm fusion‐RP column (4.6 × 150 mm) (Phenomenex Inc., Torrance, CA) was used with eluent A 0.1% (v/v) formic acid in water and eluent B 0.1% (v/v) formic acid in methanol. The detection range was set from 500 to 2000 m/z.

##### High‐Performance Liquid Chromatography (HPLC) Analysis

Purity of peptides was analyzed using an Agilent HPLC system (Agilent, Santa Clara, CA, USA) consisting of a flexible pump (G7104C), a vial sampler (G7129C), a multicolumn thermostat (G7166A) with a Quick‐connect heat exchanger (G7116−60051), and a VWD detector (G7114A). Separation was performed on a Zorbax 300 SB CN column (150 × 4.6 mm), coupled with a Security guard column (Phenomenex) at 22 °C. MI‐peptides were eluted with a linear gradient of 5 − 100% eluent B over 30 min, flow = 0.5 mL min^−1^ (eluent A = water + 0.1% TFA, eluent B = CAN + 0.1% TFA). Peptides were eluted using a linear gradient from 10% to 100% eluent B over 9 min, followed by 2 min at 100% eluent B. For the peptides CPBA‐NQEQVSPL‐PEG(6)‐azidohomoalanine and its fluorescent analog, the flow rate was set to 1 mL min^−1^, and peptides were eluted using a linear gradient from 10% to 100% eluent B over 9 min, followed by 2 min at 100% eluent B (eluent A = water + 0.1% TFA, eluent B = ACN + 0.1% TFA). Peptides were monitored at *λ* = 214 nm.

##### Cell Culture and Production of CDM

NIH 3T3 fibroblasts (RRID: CVCL_0594) (CRL‐1658; ATCC, Manassas, VA) were maintained in 100 mm culture dishes in growth medium (DMEM containing heat‐inactivated BCS (10%), penicillin G (100 U mL^−1^), and streptomycin (100 µg µL^−^^1^)) at 37 °C under CO_2_ (5%). Prior to use, the cells were seeded (16.7 × 104 cells mL^−1^, 300 µL growth medium per well) in 8‐well NuncTM Lab‐TekTM II Chamber Slides as previously reported.^[^ ^16^ ^]^ After 24 h, the medium was changed to DMEM containing heat‐inactivated FCS (10%), penicillin G (100 U mL^−1^), streptomycin (100 µg µL^−1^), and sodium L‐ascorbate (50 µg mL^−1^), and incubated for 5 d with medium exchange every 48 h. ECM components were isolated following existing protocols.^[^ ^16^ ^]^ The medium was carefully aspirated, and the cells were washed two times with PBS and subsequently incubated with ultrapure water for 15 min and a second time for 30 min, followed by three additional washing steps with PBS. After water incubation, the cells were extracted using a triton‐x ammoniac buffer (0.5% (v/v) triton‐X and 20 mM NH_4_OH in PBS, pH 7.4) for 10 min at 37 °C. After that, the extraction buffer was removed by washing with PBS five times.

##### Fluorescence Imaging of CDMs Using FL2

For visualization of fibronectin, the CDMs were first blocked using 1x Roti‐Block for 60 min at RT and washed once with PBS. A combination of Rabbit Anti‐Fibronectin antibody (F3648) (1:400 in PBS, overnight at 4 °C) and Goat Anti‐Rabbit IgG, CF633 Antibody (SAB4600141) (1:200 in PBS, 90 min at RT) was used. After washing, CDMs were incubated with 200 μL of 10 μM fluorescein boronic acid (FL2) for one hour in PBS (pH 7.4). The dye was removed, and the matrix was washed several times with one of three different buffers (carbonate/bicarbonate, pH 9.4; 1x PBS, pH 7.4; sodium‐acetate, pH 4.1). Images were taken on an Axio Observer.Z1 microscope equipped with a 63x Plan‐Apochromat 100x/1.40 Oil DIC M77 objective (Zeiss), 38 HE Green Fluorescent Reflector (*λ*ex = 450–490 nm *λ*em = 500–550 nm), 50 Cy5 Reflector (*λ*ex = 625–655 nm *λ*em = 665–715 nm) and a mercury vapor short‐arc lamp.

##### Fluorescence Imaging of CDMs with CPBA‐NQEAVSPL‐5/6‐Carboxyrhodamine 110

For visualization of fibronectin, the CDMs were first blocked using 1x Roti‐Block for 60 min at RT and washed once with PBS. A combination of Rabbit Anti‐Fibronectin antibody (F3648) (1:400 in PBS, overnight at 4 °C) and Anti‐rabbit IgG (H + L), F(ab’)2 Fragment (Alexa Fluor 647 Conjugate) 4414 (1:200 in PBS, 90 min at RT) was used. After washing, CDMs were incubated with 300 μL of 100 μM CPBA‐NQEAVSPL‐5/6‐Carboxyrhodamine 110 for three hours in carbonate/bicarbonate, pH 9.4. The dye was removed, and the matrix was washed several times with one of three different buffers (carbonate/bicarbonate, pH 9.4; 1x PBS, pH 7.4; 1x PBS, pH 8.0, and sodium‐acetate, pH 4.1). Images were taken on an Axio Observer.Z1 microscope equipped with a 63x Plan‐Apochromat 100x/1.40 Oil DIC M77 objective (Zeiss), 38 HE Green Fluorescent Reflector (*λ*ex = 450–490 nm *λ*em = 500–550 nm), 50 Cy5 Reflector (*λ*ex = 625–655 nm *λ*em = 665–715 nm) and a mercury vapor short‐arc lamp.

##### Alizarin Red S Competition Assay

The Alizarin Red S (ARS) assay previously described in Springsteen and Wang (2002) was used to characterize the boron ester formation between different monosaccharides and CPBA, as well as the CPBA‐peptide.^[^ ^19^ ^]^ The assay was performed on a TECAN reader, Infinite M Plex, set up with 96‐well plates (Thermo Scientific, Nr. 137,103). Calculation of association constants of D‐glucamine, D‐(‐)‐fructose, and D‐mannitol to CPBA and CPBA‐peptide was done as described (Figures S5–S7, Supporting Information).^[^ ^19^ ^]^

##### Myostatin Inhibition Assay

HEK SBE cells (HEK‐293 cells stably transfected with a pGl3ti vector containing a Smad binding element at the promoter of the firefly luciferase gene and a hygromycin resistance gene) were used as previously described.^[^ ^24^ ^]^ For seeding the cells in 24‐well plates, a density of 30,000 cells per well was used with the following cell culture medium (DMEM containing 4.5 g L^−1^ D‐glucose, 2 mM L‐glutamine, 10% FCS, 1% penicillin/streptomycin, and 50 μg mL^−1^ hygromycin). After 24 h, the medium was carefully removed and the cells were treated with various concentrations of MI peptides dissolved in luciferase assay medium (DMEM containing 4.5 g L^‐1^ D‐glucose, 2 mM L‐glutamine, 0.5% heat‐inactivated FCS, 1% P/S, and 50 μg ml^−1^ hygromycin). In addition, 4 nM myostatin was added to each well. After 48 h of incubation at 37 °C, 5% CO_2_, 95% rh, the medium was removed, and 100 μL of 1x Pierce Luciferase cell lysis buffer was added to each well. The plate was placed on a rocking shaker at 22–25 °C for 30 min and subsequently put on ice. The collected cell lysates were centrifuged at 13.4 rpm for 30 sec, and the supernatants were transferred to small reaction tubes and kept on ice. The total protein concentration of each sample was determined by the Bradford Protein concentration assay. For this, 5 μL of each sample was mixed with 200 μL of Bradford reagent in a transparent 96‐well plate. The absorbance was measured with a plate reader (Infinite 200 PRO, Tecan). A calibration curve was acquired using BSA standards. For the luciferase assay, 20 μL of each cell lysate was pipetted into a well of a nontransparent white 96‐well plate. The injector of the plate reader was primed with luciferase assay reagent. The plate reader was programmed to automatically inject 100 μL of the reagent into each well and immediately measure the luminescence for 10 s. As a blank, 20 μL of 1x Pierce Luciferase cell lysis buffer was used. Relative light units per μg of total protein were calculated to analyze the inhibition of myostatin by all three MI peptides.

##### Tricine‐Gel Electrophoresis

SDS‐PAGE was performed as described.^[^ ^27^ ^]^ For the separation of small molecular weight peptides, tricine‐gel‐electrophoresis (Novex 10–20% Tricine Gel, 1.0 mm × 12 well, Invitrogen by Thermo Fisher Scientific) was performed, using Mini Gel Tanks from Invitrogen under standardized conditions. The running chamber was filled with 1x Tricine‐gel running buffer (10x Tricine SDS Running Buffer, Novex by Life Technologies diluted in Mili‐Q water). Prior to loading the samples, the gel‐pockets were rinsed with 1x running buffer. Subsequently, 20 µL of the samples (diluted 1:1 with 2x sample buffer) or 5 µL of the protein ladder (SeeBlue pre‐stained, protein standard, Invitrogen by Thermo Fisher Scientific) were loaded. The gel was run at a constant voltage and current of 100 V, 3000 mA, and 350 W for 2 h. For staining, the gel was incubated with either Coomassie brilliant blue solution or Alizarin Red S solution (6 mg ARS/50 mL diluted in 10% 10x ws‐buffer, 20% methanol, 70% Mili‐Q water) for 30–60 min. The gel was destained with demineralized water or destaining buffer (10% 10x ws buffer, 20% methanol, 70% Mili‐Q water) and captured using a Gel‐Reader from BioRad.

##### Statistics

Data was analyzed using Student's t‐test or one‐way ANOVA, followed by pair‐wise comparisons using Tukey post‐hoc test. Results were considered statistically significant at *p* ≤ 0.05 (*), and results are displayed as mean with standard deviation.

## Conflict of Interest

The authors declare no conflict of interest.

## Supporting information

Supplementary Material

## Data Availability

The data that support the findings of this study are available from the corresponding author upon reasonable request.
