# Supplementary material for: Dynamic Anchoring of Peptides to the Extracellular Matrix Enabled by Boronic Acid
Source: Chembiochem. 2025 Nov 24;26(24):e202500569. doi: 10.1002/cbic.202500569 (PMC12703457; doi:10.1002/cbic.202500569)
Supplement: Supplementary file 1 — Supplementary Material [file CBIC-26-e202500569-s001.pdf]

Supplementary Information:

Dynamic Anchoring of Peptides to the Extracellular Matrix enabled by Boronic Acid

Martin Mergenthaler[a]#, Eduardo Merino Asumendi[a]#, Andreas Höpfel[a], Marcus Gutmann[a], Lorenz Meinel [a,b], Tessa Lühmann*[a]

[a] Martin Mergenthaler, Eduardo Merino Asumendi, Dr. Marcus Gutmann, Prof. Dr. Dr. Lorenz Meinel, Prof. Dr. Tessa Lühmann, Universität Würzburg, Institut for Pharmacy and Food Chemistry, Am Hubland, 97074 Würzburg, Germany, E-mail: tessa.luehmann@uni-wuerzburg.de

[b] Prof. Dr. Dr. Lorenz Meinel, Helmholtz Institute for RNA-Based Infection Research (HIRI), Helmholtz Center for Infection Research (HZI), 97080 Würzburg, Germany

#M.M. and E.M.A. contributed equally to this work.

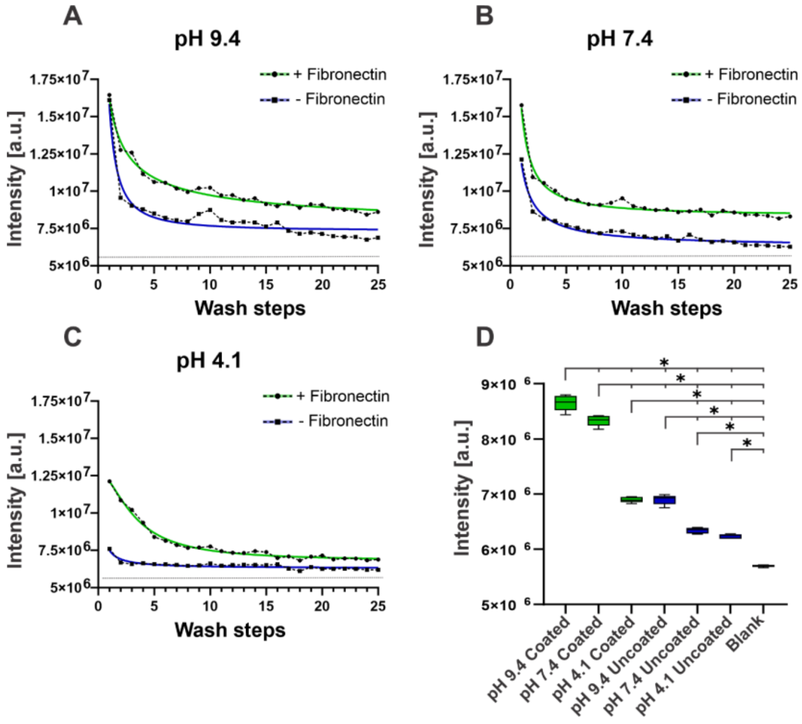

Figure F1. *Binding of FL2 to Fibronectin. The dotted grey line in A, B, and C represent the average fluorescence intensity of the untreated empty wells. The green curve represents data from wells with immobilized fibronectin while the purple curve represents wells without immobilized fibronectin. Different buffers were used for the wash steps; (A) carbonate/bicarbonate buffer pH 9.4, (B) 1x PBS pH 7.4, (C) acetic buffer pH 4.1. (D) Binding of Fibronectin. The color of the boxplots represents fibronectin-coated wells (green) and fibronectin-uncoated wells (blue). Data are shown as mean ± STDEV (n = 3) with $p \leq 0.05$ (*).*

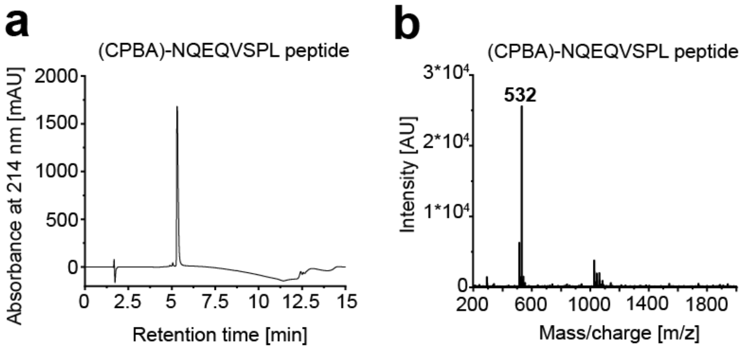

Figure S2: *(A) RP-HPLC from CPBA labelled peptide and (B) LC-MS-MS analysis.*

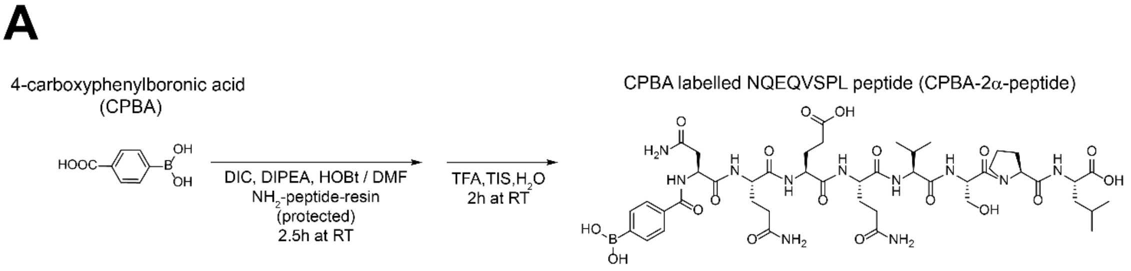

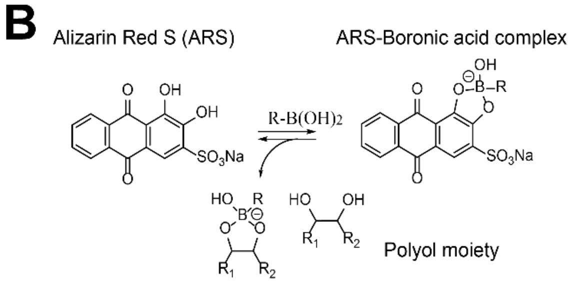

$$\frac{1}{\Delta I_f} = (\Delta kp_0 I_0 K_{eq1})^{-1} \frac{1}{[L]} + (\Delta kp_0 I_0)^{-1} \qquad (1)$$

$$\frac{[S_0]}{P} = \frac{K_{eq1}}{K_{eq}} Q + 1 \qquad (2)$$

$$P = [L_0] - \frac{1}{QK_{eq1}} - \frac{1}{Q+1} \qquad (3)$$

$$Q = \frac{[I]}{[IL]} \qquad (4)$$

Figure S3: *(A) Scheme of the chemical labeling of the NQEQVSPL peptide (CPBA-2α-peptide) with 4-carboxyphenyl boronic acid (CPBA). (B) Scheme of the mechanisms and formulas upon the ARS competition assay is based (44). I=indicator (ARS), I0=total indicator (ARS), L=ligand (boronic acid), S=substrate (monosaccharide), S0=total substrate (monosaccharide), Keq1=association constant of ARS and boronic acid, Keq=association constant of monosaccharide and boronic acid, If=fluorescent intensity, Δkp0=constant derived from the intrinsic fluorescence and the laser power.*

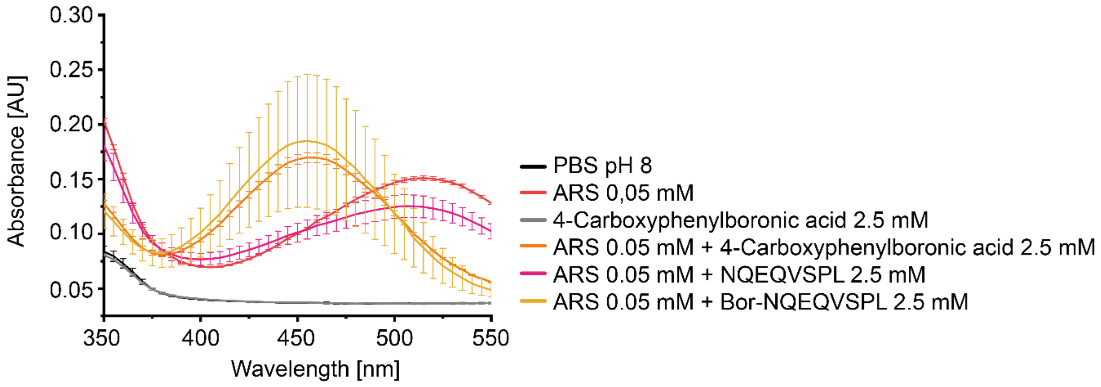

Figure S4: *Absorbance reference spectra of ARS, CPBA, the NQEQVSPL peptide, CPBA-2α-peptide and absorbance spectra after interaction with ARS at pH 8.0.*

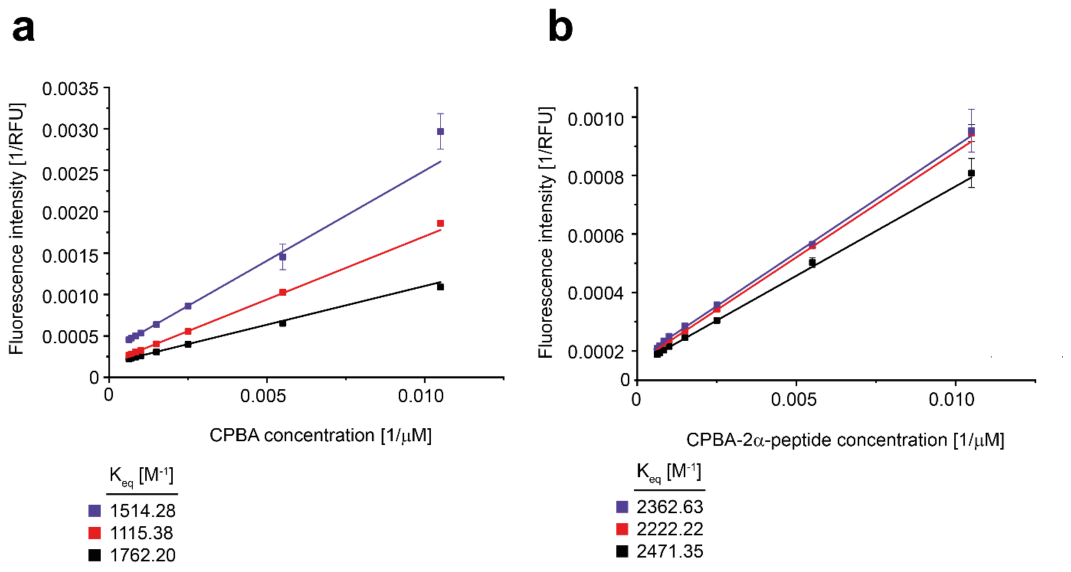

Figure S5: *(a) [1/RFU] vs [1/μM] plot to calculate the CPBA associations constant K_{eq1} to ARS. (b) [1/RFU] vs [1/μM] plot to calculate the CPBA-2α-peptide associations constant K_{eq1} to ARS.*

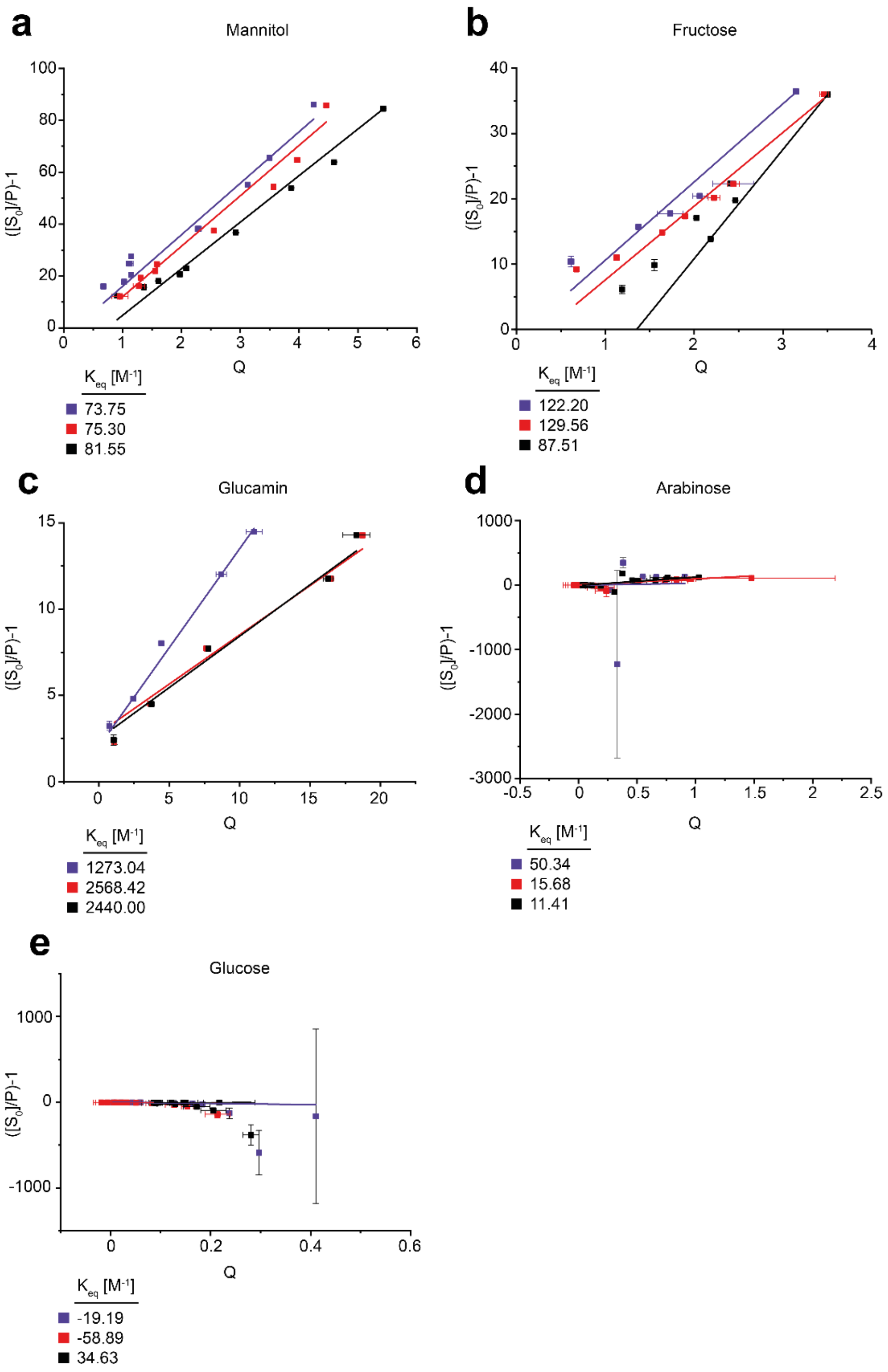

Figure S6: *[(S_0/P)-1] vs [Q] plot to calculate the CPBA associations constant K_{eq} to different monosaccharides. (a) Plot for association constant of D-mannitol to CPBA. (b) Plot for association constant of D-(-)-fructose to CPBA. (c) Plot for association constant of D-glucamine to CPBA. (d) Plot for association constant of L-(+)-arabinose to CPBA. (e) Plot for association constant of D-(α)-glucose to CPBA.*

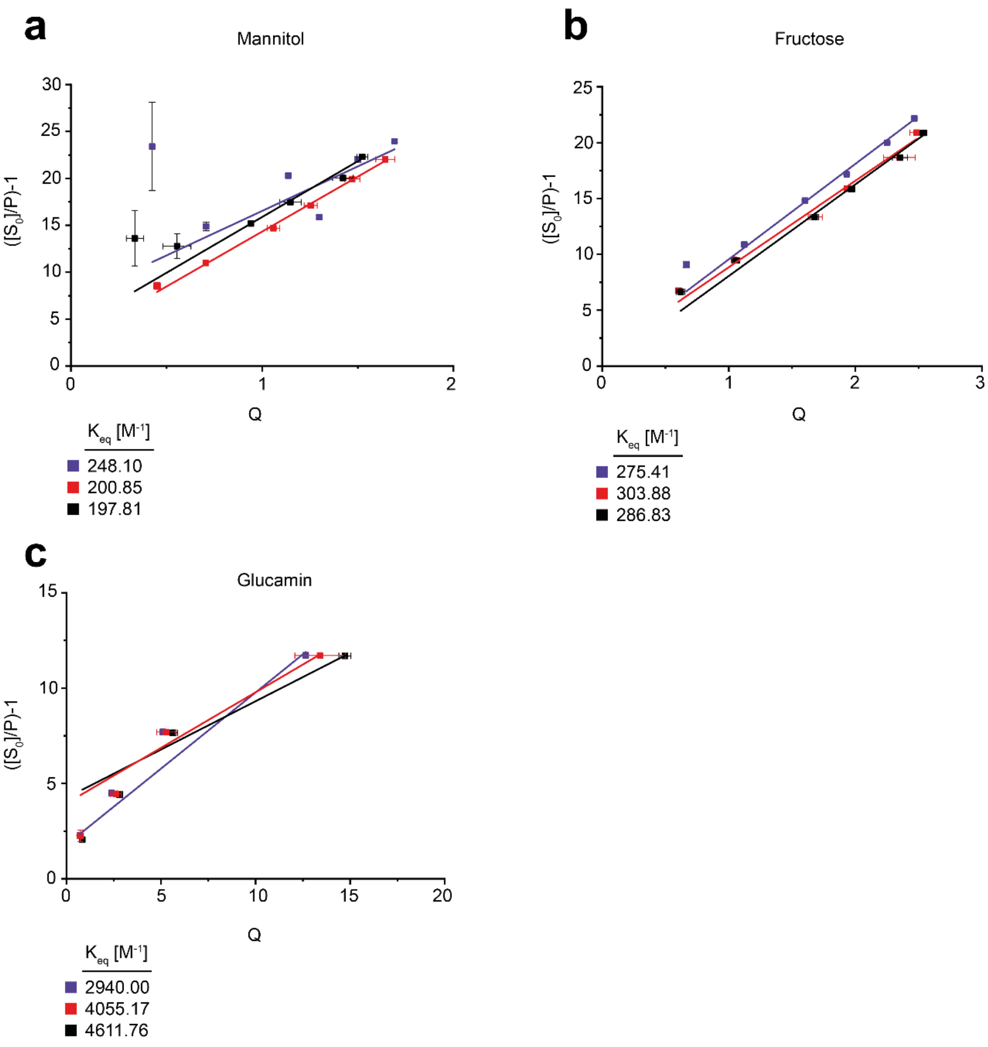

Figure S7: *[(S₀/P)-1] vs [Q] plot to calculate the CPBA-2α-peptide associations constant* K_{eq} *to different monosaccharides. (a) Plot for association constant of D-mannitol to CPBA-2α-peptide. (b) Plot for association constant of D-(-)-fructose to CPBA-2α-peptide. (c) Plot for association constant of D-glucamine to CPBA-2α-peptide.*

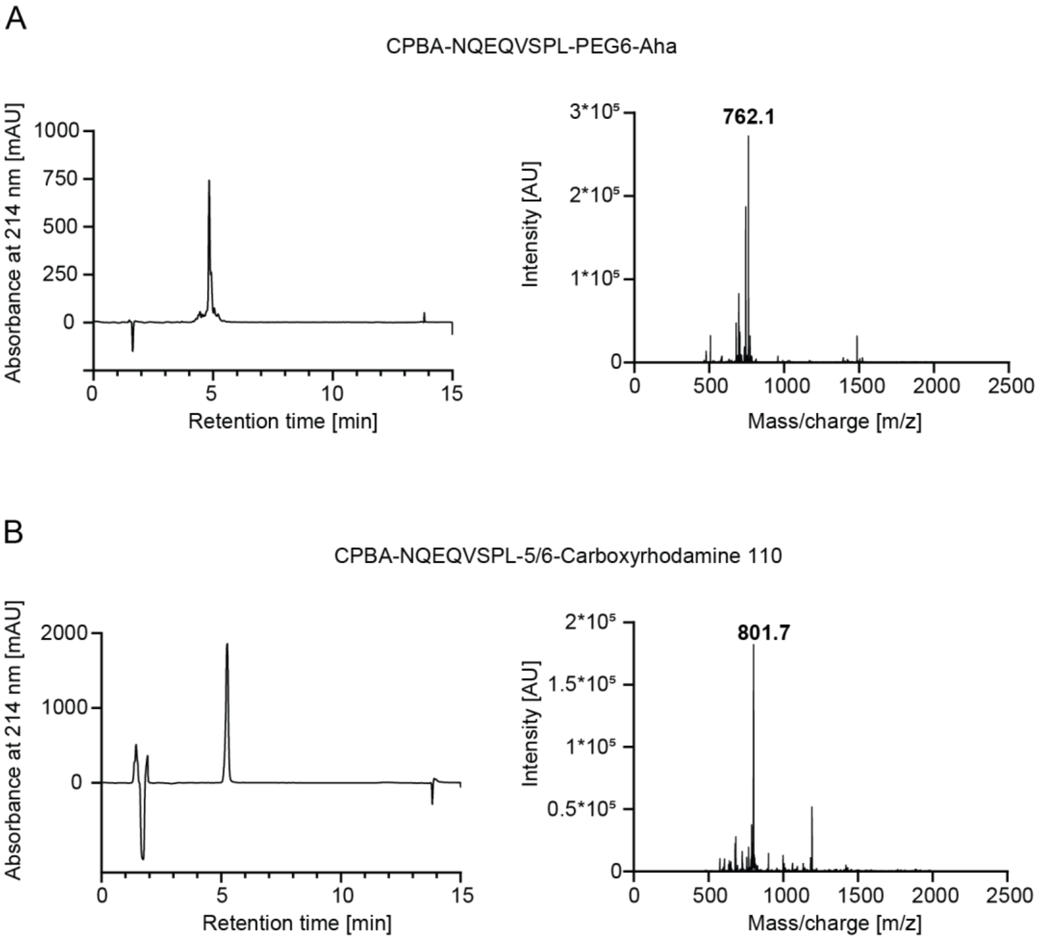

Figure S8: *(A) RP-HPLC and LC-MS/MS for the CPBA labelled peptide designed for fluorophore conjugation. (B) RP-HPLC and LC-MS/MS for the CPBA labelled peptide with Carboxyrhodamine 110.*

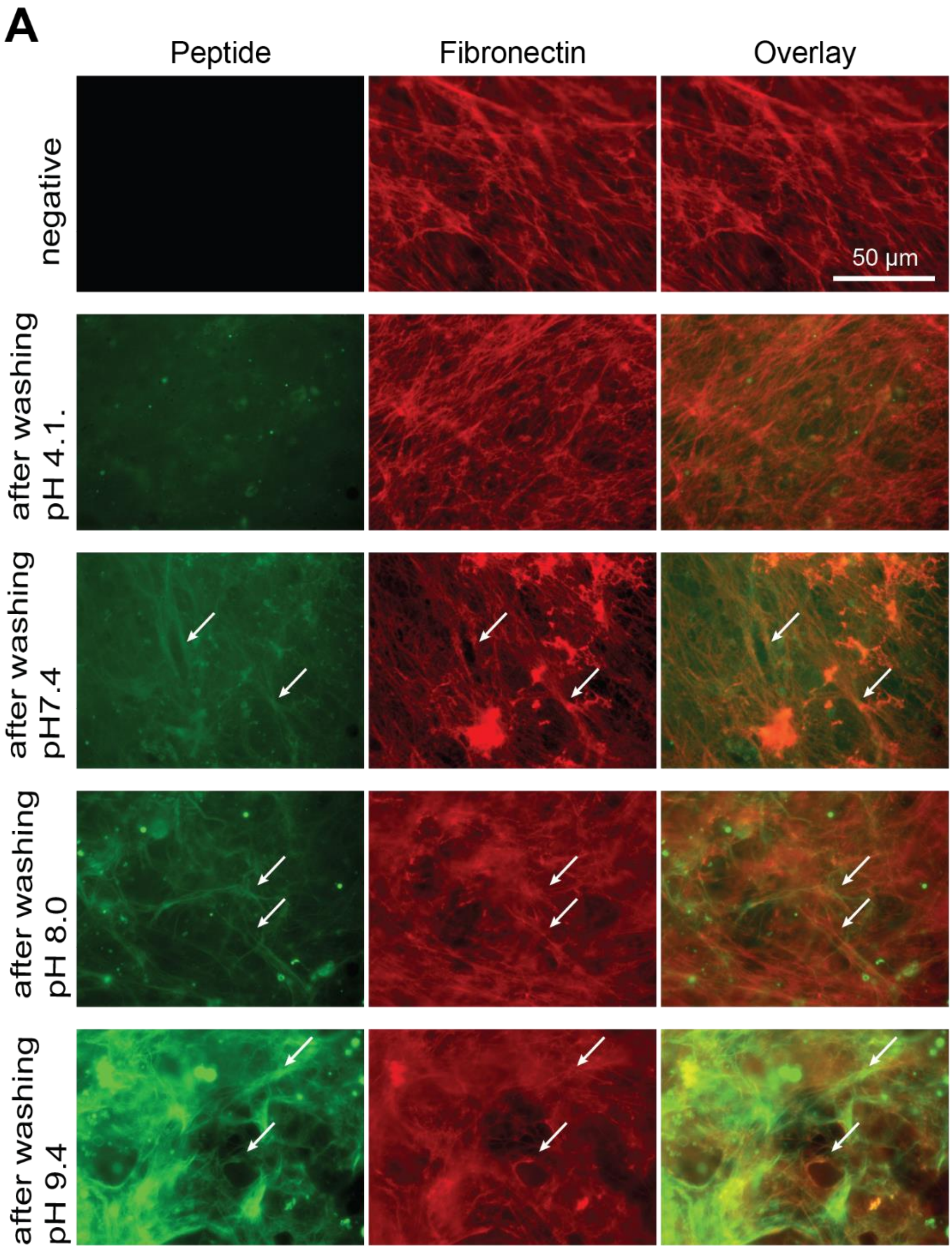

Figure S9: *(A) Fluorescent microscopy of CDM. Green channel: Staining with CBPA-NQEQVSPL-5/6-Carboxyrhodamine 110 is shown. Red channel: Staining with a primary antibody (rabbit; Anti-fibronectin) and secondary antibody (Anti-Rabbit IgG, AF647) is shown. Third channel: Both channels are merged. The negative control was only stained with the antibodies. Every CDM with the CPBA-Peptide was washed several times with either pH 4.1, pH 7.4, pH 8.0 or pH 9.4.*

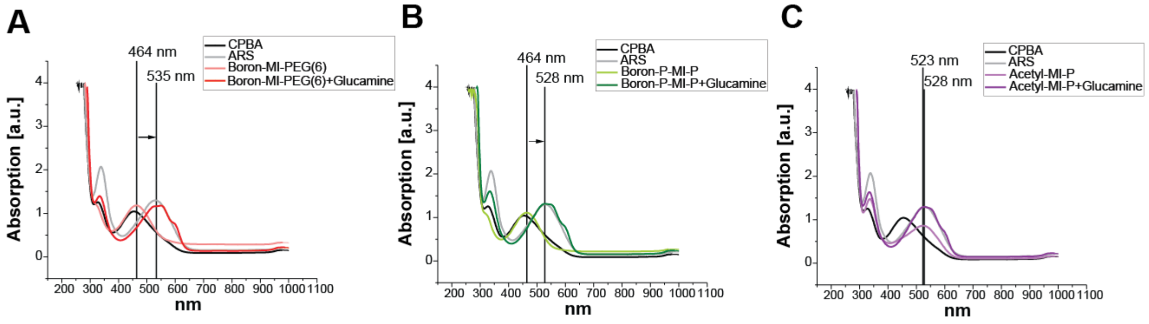

Figure S10: *UV-VIS spectra of various peptides bound to ARS or glucamine. CPBA was used as a positive control, while ARS serves as a negative control. (A) UV-VIS-spectra of the peptide CPBA-MI in the presence of ARS- or glucamine. (B) UV-VIS-spectra of the peptide CPBA-PEG-MI in the presence of ARS- or glucamine. (C) UV-VIS-spectra of the peptide Acetyl-MI in the presence of ARS- or glucamine.*

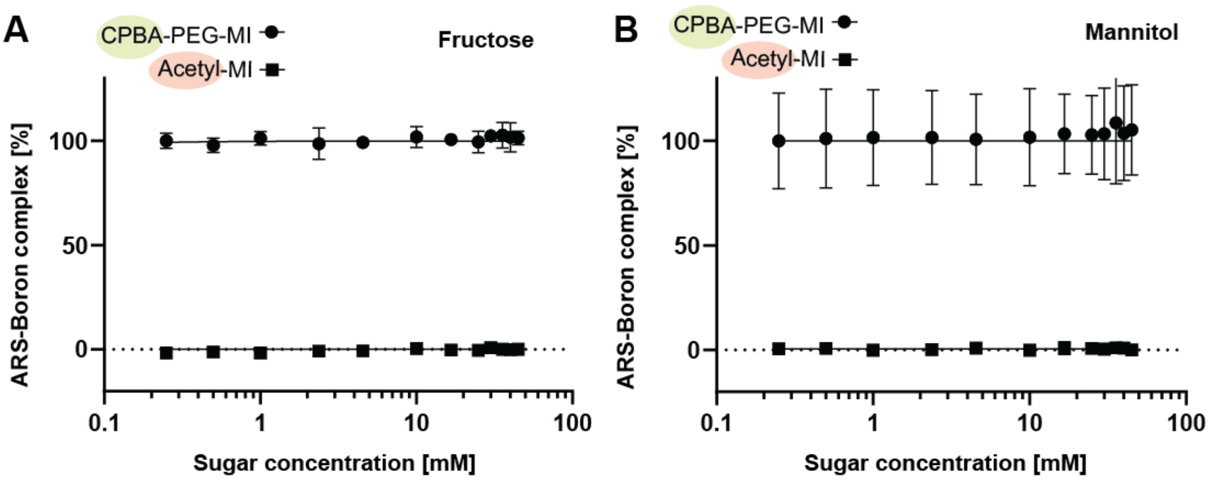

Figure S11: *Fluorescence decay at 572 nm measured in an ARS competition assay with CPBA-PEG-MI and the negative control acetyl-MI in the presence of rising concentrations of fructose (A) and mannitol (B) as model saccharides at pH 7.4. Data are shown as mean ± STDEV (n = 3).*
